# Spectrum of pneumothorax/pneumomediastinum in patients with coronavirus disease 2019

**DOI:** 10.5339/qmj.2021.41

**Published:** 2021-09-07

**Authors:** Anirban B Adhikary, Udhyachander R, Nupur B Patel, Vadhan Prasanna S, Priyanka Boruah, Saurabh Chandrakar

**Affiliations:** Department of Anaesthesiology and Intensive Care Medicine, All India Institute of Medical Sciences, Rishikesh, India drsaurabh6587@gmail.com

**Keywords:** COVID-19, pneumothorax, pneumomediastinum, PEEP, pneumopericardium

## Abstract

Background: Spontaneous pneumothorax/pneumomediastinum is an uncommon complication of coronavirus disease 2019 (COVID-19). Herein, we describe the clinical spectrum and outcomes of COVID-19-associated pneumothorax/pneumomediastinum in critical care settings.

Materials and methods: We hereby present a case series of 12 patients who tested positive for COVID-19 and developed air leak injuries in critical care settings in a tertiary care center in Northern India. Infection with severe acute respiratory syndrome coronavirus-2 was confirmed by nasal/oropharyngeal swab testing using real-time reverse-transcription polymerase chain reaction test. The clinical spectrum and outcomes of these patients were assessed. Each case has been presented as a brief synopsis.

Results: The onset of pneumothorax/pneumomediastinum varied from 11 to 28 days after the occurrence of initial symptoms and caused worsening of respiratory parameters in most patients. Of the 12 patients, eight were males who developed air leak injuries. One patient was a current smoker, and three patients had underlying lung disorders. Two patients with spontaneous breathing were managed conservatively. All intubated patients who developed air leak injuries died (100% mortality rate).

Conclusion: Pneumothorax/pneumomediastinum is a rare and life-threatening complication in mechanically ventilated patients with COVID-19. Further research is needed to understand the pathophysiology behind the development of air leak injuries in patients with COVID-19.

## Introdcution

After the outbreak of coronavirus disease 2019 (COVID-19) in Wuhan, China, clinicians are observing a myriad of presentations of this disease. Although a considerable amount has been discussed in the literature regarding ground-glass attenuation, septal thickening, and lung infiltrate in severe acute respiratory syndrome coronavirus-2 (SARS-CoV-2) infection, pneumothorax, pneumomediastinum, and pneumopericardium are rare presentations with an incidence of less than 2%.^1–3^

High positive end-expiratory pressure (PEEP), barotrauma, and undiagnosed bullous lung disease have taken all the blame for this air leak in patients with COVID-19.^4,5^ In this article, we present cases of 12 patients with COVID-19 from a Northern Indian tertiary care hospital who developed air leak injury and had varying PEEP levels. These patients included those who were spontaneously breathing and those who were mechanically ventilated. This case series has been approved by the institutional ethics committee (IEC/20/655), and informed consent was obtained from all patients.

## Case Series

### Case 1: Pneumothorax on noninvasive ventilation [NIV]

**Figure 1.**
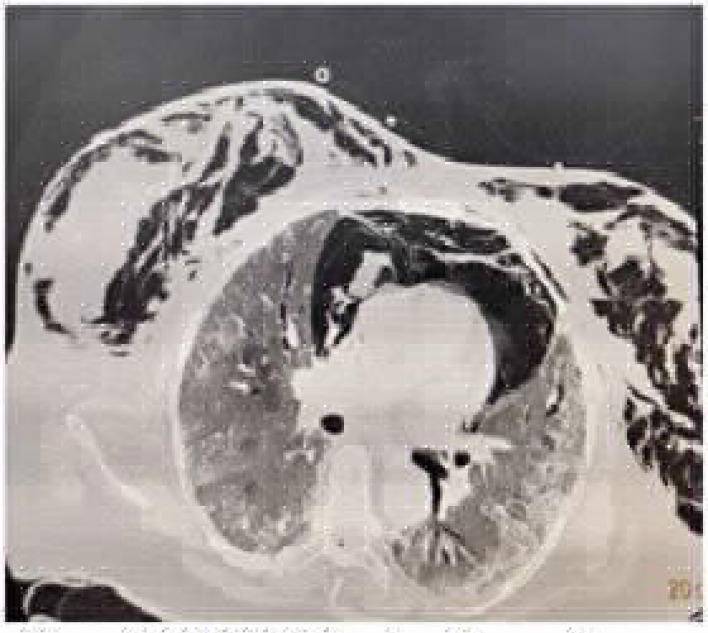
CASE 1: Tension Pneumothorax with surgical emphysema

A 65-year-old female patient presented to the emergency department with complaints of worsening shortness of breath since 5 days, low-grade fever since 7 days, and dry cough since 12 days. She had diabetes and hypertension since 12 years and was receiving irregular medications. On admission, the patient had a respiratory rate of 30 breaths per minute and oxygen saturation of 80% in room air, which improved to 89% with 15 L/min on reservoir bag. Reverse-transcription polymerase chain reaction (RT-PCR) of oropharyngeal and nasal swabs were positive for COVID-19, and subsequently, she was transferred to the critical care unit. As per the institutional protocol, antivirals, steroids, and anticoagulants were initiated. However, the patient's condition worsened clinically, and she required NIV support (fraction of inspired oxygen [FiO_2_]: 0.6, pressure support [PS]: 10, PEEP: 6) for the next 4 days. Gradually, her oxygenation status deteriorated, and she developed subcutaneous emphysema over the chest. Later, she was intubated. High-resolution computed tomography (HRCT) revealed that the patient had mediastinal emphysema with ground-glass opacity, for which an interventional radiologist inserted a mediastinal drain and subsequently, her oxygen requirement reduced (Figure 1). However, the patient developed secondary bacterial pneumonia and died on day 42 of illness due to worsening hypoxemia.

### Case 2: Pneumomediastinum and pneumothorax on low-flow oxygen

**Figure 2.**
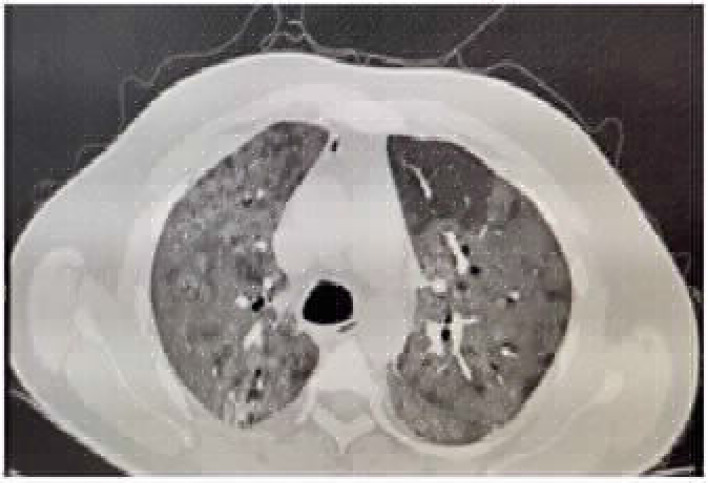
CASE 2: Pneumopericardium in spontaneously breathing patient

A 45-year-old male patient with no known comorbidity was admitted to the hospital with worsening shortness of breath since 7 days (deteriorated to Modified Medical Research Council [MMRC] grade 3) as well as occasional low-grade fever and nonproductive cough since a day. On examination, the patient was found to have a respiratory rate of 28 breaths per minute with no use of accessory muscles and 81% saturation in room air, which improved to 89% on 0.4 FiO_2_ using the Venturi mask. The patient's RT-PCR test for COVID-19 was positive; subsequently, he was started on treatment according to institutional protocol. His chest HRCT revealed a chest computed tomography (CT) severity score (CTSS) of 40/40 with minimal bilateral pneumothorax and small pneumomediastinum (Figure 2). He was managed conservatively and then discharged on day 34 of his illness without any complication.

### Case 3: Pneumomediastinum and pneumothorax on high-flow nasal cannula

**Figure 3.**
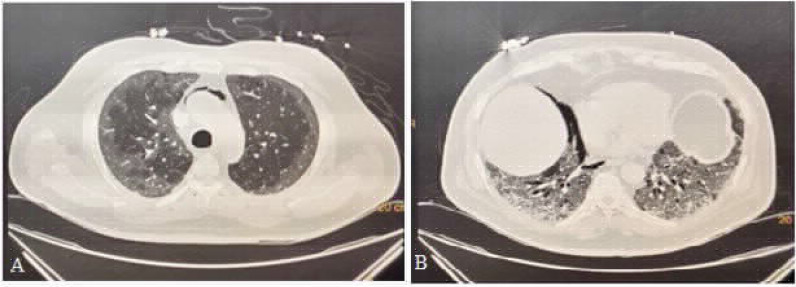
CASE 3: A: minimal pneumomediastinum, B: right Pneumothorax

A 62-year-old male patient was admitted to emergency department with a positive COVID-19 RT-PCR report from another hospital. He complained of low-grade fever and nonproductive, intermittent cough since 7 days along with shortness of breath since a day. The patient had a saturation of 84% on 15 L/min oxygen therapy on arrival and was started on high-flow nasal cannula oxygen delivery (flow: 60 L/min, FiO_2_: 1.0). His baseline HRCT revealed a CTSS of 36/40 along with pneumomediastinum and minimal right-sided pneumothorax, which was managed conservatively (Figure 3a and Figure 3b). He then received treatment as per the institutional protocol. However, his clinical condition worsened, and he required mechanical ventilation on day 20 of illness. Despite aggressive therapy, he succumbed to his illness on day 29 of illness.

### Case 4: Pneumomediastinum on reservoir bag (15 L/min)

**Figure 4.**
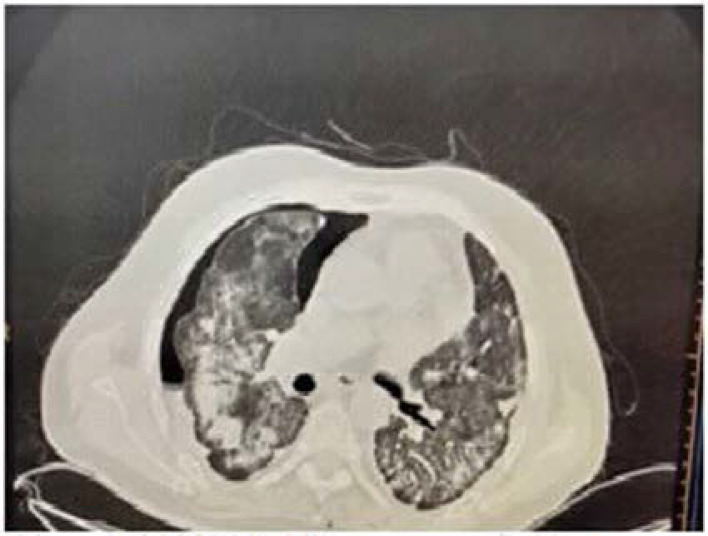
CASE 4: Right pneumomediastinum with pneumothorax

A 60-old hypertensive male patient was admitted with moderate to high-grade fever along with sore throat for 5 days that was associated with cough and worsening breathlessness since the last 2 days. As his COVID-19 RT-PCR report was positive, he received treatment as per the institutional protocol. His baseline HRCT revealed a CTSS of 32/40 with right-sided pneumothorax, which was managed conservatively (Figure 4). Subsequently, his clinical condition improved, and he was discharged on day 42 of his illness

### Case 5: Bilateral pneumothorax with severe acute respiratory distress syndrome [ARDS]

**Figure 5.**
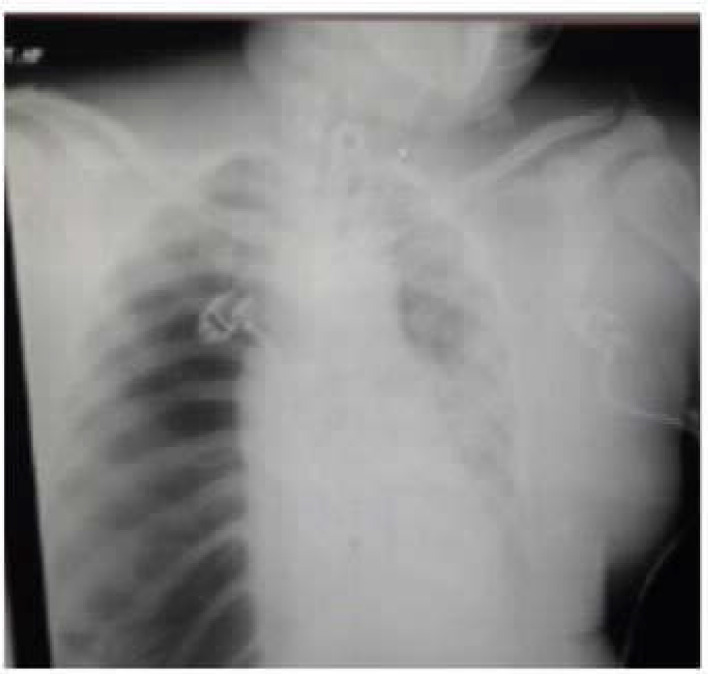
CASE 5: Right Pneumothorax in chest X-ray

A 30-year-old female patient with no known comorbidity was admitted to our hospital with low sensorium (Glasgow Coma Score = 3/15; brain noncontrast computed tomography showed diffuse axonal injury) after road traffic accident, for which she was electively ventilated and conservatively managed. Gradually, the patient developed severe ARDS, for which lung-protective ventilation was initiated. Owing to high clinical suspicion, repeat sampling of nasal and oropharyngeal swabs was conducted, and the RT-PCR revealed positivity for COVID-19. Chest X-ray showed bilateral pneumothorax, for which a bilateral intercostal drain was inserted (Figure 5). Her oxygen requirement worsened progressively, and she additionally developed refractory shock; subsequently, she succumbed to her illness on day 30.

### Case 6: Unilateral pneumothorax with moderate ARDS

**Figure 6.**
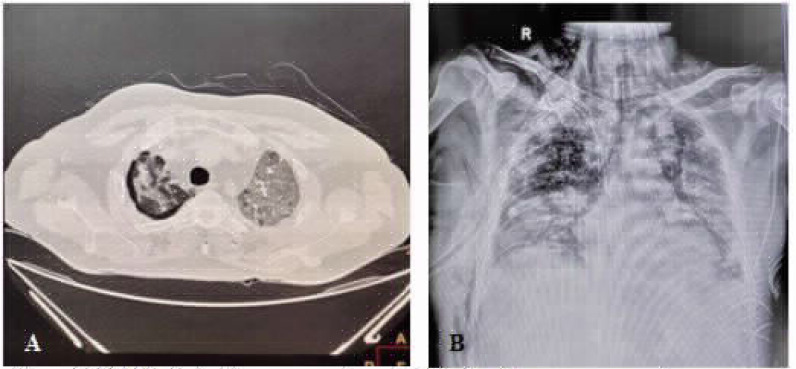
CASE 6: A: right pneumothorax in CT, B: subcutaneous emphysema with drain in situ

A 43-year-old male patient with no known comorbidity was admitted to the emergency department with increasing cough and shortness of breath since 5 days. His oxygen saturation at room air was 81%, which improved to 89% with 15 L/min reservoir bag. His HRCT revealed a CTSS of 39/40 with ground-glass opacity. The patient gradually experienced increased difficulty in breathing and was noncompliant to NIV. Thus, he was intubated on day 10 of illness and received lung-protective ventilation. Lung ultrasound and chest X-ray revealed right pneumothorax, for which a right-sided intercostal drain was inserted (Figure 6a and Figure 6b). However, the patient's clinical condition worsened, and he succumbed on day 26 of his illness.

### Case 7: Pneumomediastinum in a patient with chronic obstructive pulmonary disease [COPD]

**Figure 7.**
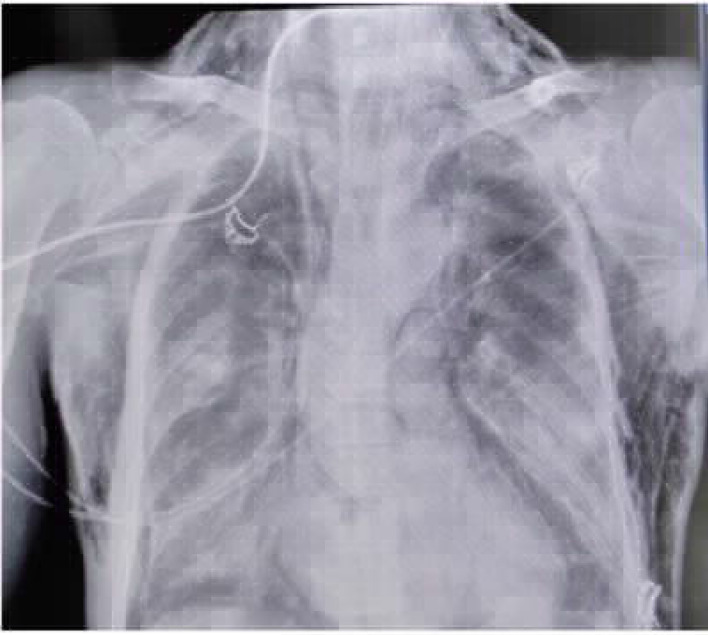
CASE 7: bilateral pneumothorax with mediastuinal emphysema

A 77-year-old male patient was admitted with generalized weakness and progressive shortness of breath for 7 days, for which NIV was initiated in the emergency department. He was a chronic alcoholic and smoker and had hypertension and COPD, for which he was irregularly receiving medication. The next day, he tested positive for COVID-19 via an RT-PCR test. HRCT revealed that the patient had emphysematous bullae with a CTSS of 36/40. On day 14 of illness, the patient's oxygen saturation decreased to 81% on FiO_2_ of 1.0 with NIV support. Thus, he was intubated and ventilated as per lung-protective ventilation strategy. However, the patient developed subcutaneous emphysema over the chest and face, and a chest X-ray was suggestive of mediastinal emphysema; thus, a drain was inserted (Figure 7). However, his shock and oxygen requirement worsened, and he succumbed on day 22 of his illness.

### Case 8: Pneumopericardium in a young male on mechanical ventilation

**Figure 8.**
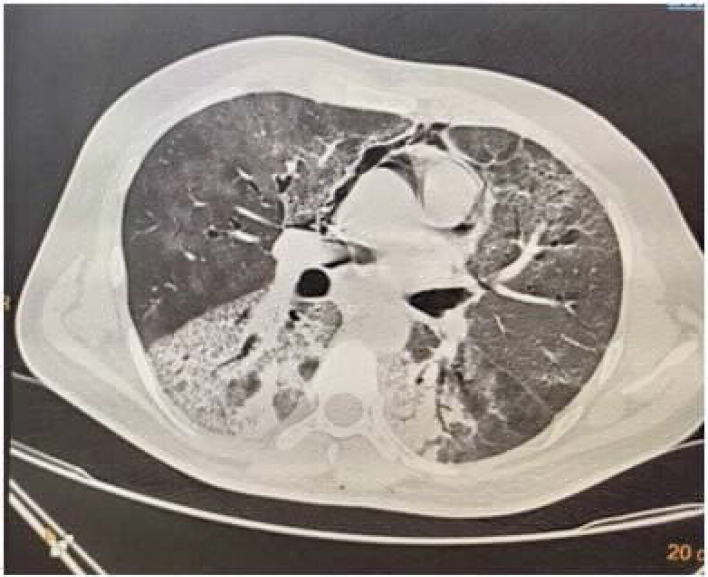
CASE 8: Pneumopericardium extending along the great vessels into the left carotid sheath

A 25-year-old male patient with no known comorbidity was admitted with complaints of low-grade fever since 7 days along with shortness of breath for 3 days. In the emergency department, the patient had oxygen saturation of 70% at room air, which improved to 94% on NIV (FiO_2_: 0.7, PEEP: 7, PS: 10). HRCT revealed CTSS of 37/40 with ground-glass opacity. Subsequently, the patient was intubated on day 7 of hospitalization. Computed tomography pulmonary angiogram (CTPA) showed no features of pulmonary embolism; however, mild pneumopericardium was observed, which was managed conservatively (Figure 8). Owing to the worsening of his illness, he succumbed on day 24.

### Case 9: Pneumothorax with interstitial lung disease

**Figure 9.**
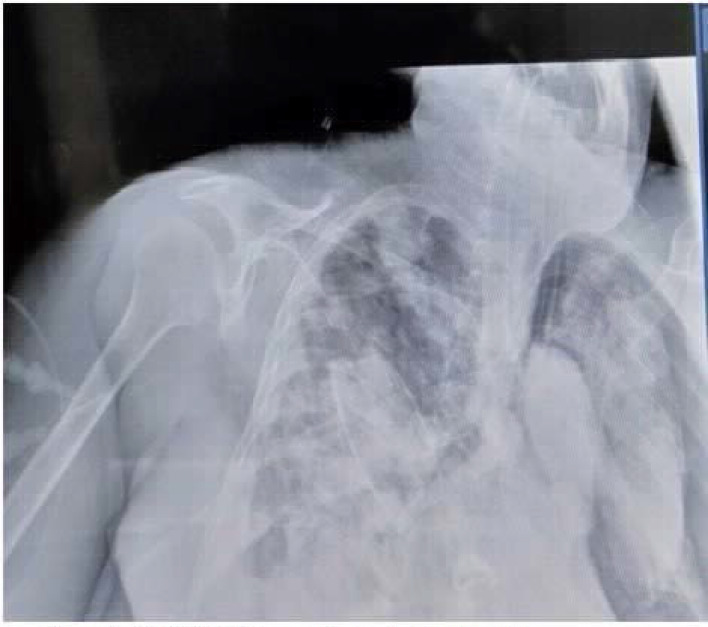
CASE 9: left pneumothorax in chest X-ray

A 52-year-old female was admitted with a history of intermittent fever since 10 days along with nonproductive cough for 7 days. She had diabetes, hypertension, and interstitial lung disease. In the emergency department, he had respiratory distress with an oxygen saturation of 60% at room air, and thus, she required emergency intubation. Chest X-ray confirmed the presence of a left-sided pneumothorax, for which an intercostal drain was inserted (Figure 9). The patient developed features of myocarditis with cardiogenic shock along with severe ARDS, and she subsequently succumbed on the 27^th^ day of illness.

### Case 10: Pneumothorax with acute cor pulmonale

A 38-year-old male patient, with a recent history of diabetes and hypertension, was admitted for COVID-19 confirmed using an RT-PCR test. He presented with low-grade fever for 7 days, cough and shortness of breath for 3 days, and sore throat with malaise for 2 days. Initial assessment revealed an oxygen saturation of 81% at room air; thus, he received treatment per the institutional protocol and NIV (FiO_2_: 0.6, PS:10, PEEP: 7) as well as awake proning. However, his oxygen requirement gradually increased, and he required mechanical ventilation (FiO_2_: 1.0, tidal volume: 320 mL, PEEP: 12, respiratory rate: 32/min) and proning. CTPA findings were not suggestive of pulmonary embolism. His CTSS was 40/40 with right pneumothorax, for which an intercostal drain was used. Later, he developed a bronchopleural fistula. Echocardiography was suggestive of significant right ventricular dysfunction. However, his hypoxemia and shock worsened, and he succumbed on day 21 of illness.

### Case 11: Pneumomediastinum in a patient with COPD and secondary bacterial pneumonia

**Figure 10.**
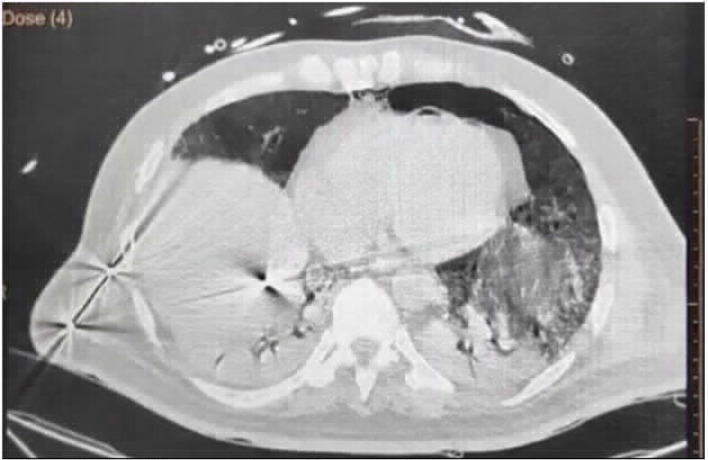
CASE 11: left pneumothorax with pneumomediastinum

A 52-year-old alcoholic male patient with diabetes and COPD, who was on irregular treatment, was admitted with worsening shortness of breath (MMRC grade: I to IV) since the last 15 days. Upon his presentation in the emergency department, urgent intubation was performed. At admission, his CTSS was 35/40 with ground-glass opacity and no documented bullae. After 15 days of stay in the intensive care unit, his respiratory parameters worsened and so did the characteristics of secondary bacterial pneumonia (FiO_2_: 1.0, PEEP: 8, tidal volume: 340 mL, respiratory rate: 18 breaths/min). Repeat HRCT on day 30 showed new pneumomediastinum with the consolidation of the right lower lobe and a CTSS of 38/40 (Figure 10). Subsequently, he underwent mediastinal drain insertion. However, his hypoxemia did not improve, and he died on day 37 of his illness.

### Case 12: Pneumopericardium on NIV

**Figure 11.**
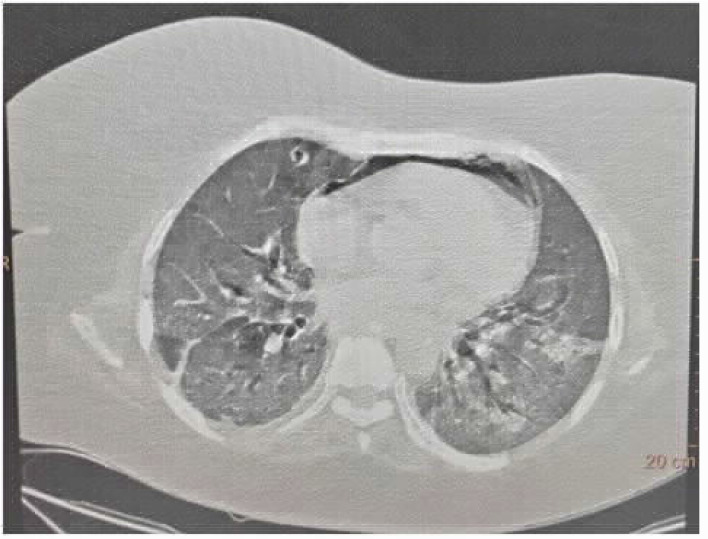
CASE 12: pneumopericardium on CT image

A 58-year-old female patient was admitted to the emergency department with loose stools, malaise, and sore throat since 2 days. She had diabetes since 10 years and was regularly receiving medications. Upon presentation in the emergency department, her oxygen saturation was 84% at room air, with the use of accessory muscles, and her urine tested negative for ketones. The patient's COVID-19 RT-PCR report came positive. Subsequently, her oxygen requirements from the reservoir bag increased, and she required noninvasive ventilator support (FiO_2_: 0.6, PS: 12, PEEP: 7). HRCT revealed multiple patchy areas of ground-glass attenuation with interlobular septal thickening in bilateral lungs with a CTSS of 36/40. Pneumopericardium was also noted with a maximum thickness of 12.7 mm extending into the arch of the aorta, which was managed conservatively (Figure 11a and Figure 11b). However, she developed acute kidney injury with refractory metabolic acidosis and shock. Thus, hemodialysis was initiated, along with treatment using vasoactive agents. However, she succumbed on the 22nd day of illness.

The clinical profile and baseline investigations of all patients have been described in Table 1 and Table 2, respectively.

## Discussion

During our clinical experience with COVID-19 from August to September 2020, we encountered 32 cases of subcutaneous emphysema, pneumothorax, and pneumomediastinum in 146 critically ill patients requiring mechanical ventilator support, with barotrauma as an insulting agent. However, this article describes a series of 12 patients who developed pneumothorax, pneumomediastinum, or pneumopericardium with varying PEEP level; these patients also include those who were spontaneously breathing.

In our study, the onset of pneumothorax/pneumomediastinum/pneumopericardium varied from 11 to 28 days after the occurrence of initial COVID-19 symptoms and led to deterioration in most patients. No patients had any bullous disorder/pneumatocele at the time of admission. Pulmonary embolism is also a common presentation during the initial presentation of COVID-19.^6^ In sudden-onset respiratory insufficiency in patients with COVID-19, repeated clinical and sonological/radiological examination helps in differentiating pulmonary embolism from air leak injuries.

In the present study, only one patient was a current smoker; however, three patients had underlying lung disorders. Similar to study published in China by Chen et al., in our study, being a male was a risk factor for air leak injuries.^7^

In our study, more than 60% of cases developed air leak injuries spontaneously or with lower requirements of positive pressure ventilation. The relation between air leak injuries and disease process is complex and not completely understood. Postmortem analysis of patients who succumbed to COVID-19 revealed diffuse alveolar damage with fibromyxoid exudates, desquamation of pneumocytes, hyaline membrane formation, and lymphocyte infiltration in the lungs.^8,9^ The pathological features of our patients were very similar to those of patients with SARS and Middle East respiratory syndrome (MERS).^10,11^ However, the incidence of pneumothorax in patients with MERS varied from 15% to 30%.^12^ Multiple studies have demonstrated early cyst formation and extensive alveolar destruction in the area of airspace disease, which in turn can result in air leakage and interstitial emphysema.^13–16^ Apart from that, severe endothelial injury and intussusceptive angiogenesis^17^ were also documented in lung autopsy specimens. It is possible that these complex molecular and pathogenic changes can predispose patients to air leak injuries.

Management of air leak injury varies from conservative treatment to the use of drain placement, and in refractory cases, surgical intervention is opted.^18^ In our study, patients who were not on positive pressure ventilation were managed conservatively. For those requiring invasive ventilation, an intercostal drain was placed, followed by a mediastinal drain. One patient developed nonresolving pneumothorax and was scheduled for surgical intervention; however, he was hemodynamically unstable and developed severe acidosis and died before any intervention.

Our study had few limitations. Serial chest HRCT was not performed in stable patients owing to limited resources and improving clinical condition. It would have been interesting to obtain postmortem findings of the patients presented in this case study to determine the pathological changes in lung parenchyma; however, local institutional policies did not permit this.

## Conclusion

This study reports a series of patients with COVID-19 pneumonia with hypoxemia who required varying levels of positive pressure ventilation and developed air leak injuries; thereby, the study contributes to the existing literature. Meanwhile, we highlight that these injuries can also be developed in patients with spontaneous breathing or in those requiring noninvasive ventilation.

### Author contributions

ABA: Patient treatment, data collection, conception, substantial contribution to drafting manuscript, final approval

UR: Patient treatment, data collection, revising the draft

NBP: Patient treatment, data collection, conception, final approval

VSP: Patient treatment, data collection, revising the draft, final approval

PB: Patient treatment, data collection, revising the draft, final approval

SC: Patient treatment, data collection, conception, substantial contribution to drafting manuscript, final approval

The authors declare that they do not have a conflict of interest or financial relationship regarding this manuscript.

No funding has been obtained for this report.

### Availability of data and material

The datasets used and analyzed in the study are available from the corresponding author on reasonable request.

## Figures and Tables

**Table 1:** The clinical profile of patients

Variables/case numbers	CASE 1	CASE 2	CASE 3	CASE 4	CASE 5	CASE 6	CASE 7	CASE 8	CASE 9	CASE 10	CASE 11	CASE 12

Age (years)	65	45	62	60	30	43	77	25	71	38	52	58

Sex	F	M	M	M	F	M	M	M	F	M	M	F

Smoking	NO	NO	NO	NO	NO	NO	YES	NO	NO	NO	NO	NO

Comorbidities	DM	NIL	NIL	HTN	NIL	NIL	COPD	NIL	DM, HTN, ILD	DM, HTN	DM, COPD	DM, HTN

PEEP at admission (centimeters of water)	0	0	0	0	5	5	5	7	7	7	7	0

PEEP when air leak was diagnosed (centimeters of water)	6	0	0	0	12	8	8	10	10	12	8	7

Chest CT severity score (CTSS)	40/40	40/40	36/40	32/40	NOT PERFORMED	39/40	36/40	37/40	NOT PERFORMED	40/40	35/40	36/40

PT/PM/PPC	PM	PT	PM	PM	PT	PT	PM +bilateral PT	PPC	PT	PT	PM +PPC	PPC

Day on which respective pneumothorax/pneumomediastinum developed after the onset of illness	28	12	17	24	11	16	17	16	26	16	30	18

Intervention	Pigtail	NIL	NIL	NIL	Bilateral ICD	ICD	Bilateral ICD	NIL	ICD	ICD	Pigtail	NIL

Duration of illness (days)	42	28	29	42	30	26	22	24	27	21	37	22

Outcome	Died on day 30 after hospital admission	Discharged after 28 days in hospital	Died on day 22 after hospital admission	Discharged after 42 days in hospital	Died on day 29 after hospital admission	Died on day 21 after hospital admission	Died on day 15 after hospital admission	Died on day 17 after hospital admission	Died on day 17 after hospital admission	Died on day 14 after hospital admission	Died on day 22 after hospital admission	Died on day 20 after hospital admission


DM = Diabetes, HTN = Hypertension, COPD = Chronic obstructive pulmonary disease, ILD = Interstitial lung disease, PEEP = Positive end-expiratory pressure, CTSS = Chest CT severity score, PM = Pneumomediastinum, PT = Pneumothorax, PPC = Pneumopericardium, ICD = Intercostal drain, M = Male, F = Female

**Table 2:** Biochemical parameters of patients on admission

VARIABLES/CASE NUMBERS	CASE 1	CASE 2	**CASE 3**	CASE 4	CASE 5	CASE 6	CASE 7	CASE 8	CASE 9	CASE 10	CASE 11	CASE 12

D-dimer (ng/ml)	1555.0	>5000	894	4665	NA	893	NA	2531	>5000	878.65	>5000	>5000

Procalcitonin (ng/ml)	0.05	NA	0.10	NA	NA	>0.1	NA	0.23	NA	0.41	0.114	1.23

Interleukin-6 (pg/ml)	32.3	NA	103.60	13.7	NA	75.6	NA	86.4	NA	32.9	NA	23.7

TLC (cells/mm^3^)	9810	4717	12170	17800	11730	11770	3819	14500	10300	5250	14400	6060

Lymphocyte (%)	2.6	NA	4.3	11.13	75	1.9	17	3.6	14	8	87	77.5

CRP (mg/L)	NA	NA	7.63	NA	NA	NA	NA	134	39.6	NA	53.9	NA

Platelet (thousands/mL)	263	430	267	256.8	364	187	240	299.7	138	103	233.8	98.8

Blood urea (mg/dL)	37.3	22	48.1	50.4	26.9	34.2	42	31	59	21.3	38.9	100.9

Serum creatinine (mg/dL)	0.66	0.68	1.11	0.80	0.51	0.72	1.7	0.82	1.8	0.88	0.98	2.37

Total bilirubin (mg/dL)	1.02	0.4	0.6	0.52	0.5	0.46	5.3	1.69	0.47	.74	1.07	0.33

AST/ALT (U/L)	36/50	44/57	75/209.2	26/25.1	61/60	48/64.9	102/42	406/294	177/21	49/41	56.5/117.4	50.5/58.3

Ferritin (ng/mL)	238.6	NA	894.14	4665	NA	893	NA	2531	>5000	878.6	>5000	>5000


TLC = Total leucocyte count, CRP = C-reactive protein, AST = Aspartate amino transferase, ALT = Alanine transaminase, NA = Not available
